# Mitochondria and aging in older individuals: an analysis of DNA methylation age metrics, leukocyte telomere length, and mitochondrial DNA copy number in the VA normative aging study

**DOI:** 10.18632/aging.102722

**Published:** 2020-02-02

**Authors:** Jacopo Dolcini, Haotian Wu, Jamaji C. Nwanaji-Enwerem, Marianthi-Anna Kiomourtozlogu, Akin Cayir, Marco Sanchez-Guerra, Pantel Vokonas, Joel Schwarz, Andrea A. Baccarelli

**Affiliations:** 1 Department of Environmental Health Sciences, Mailman School of Public Health, Columbia University, New York, NY 10032, USA; 2 Department of Biomedical Sciences and Public Health, Section of Hygiene and Preventive Medicine, Medical School, Polytechnic University of Marche, Ancona, Italy; 3 Department of Environmental Health, Harvard T.H. Chan School of Public Health, Boston, MA 02115, USA; 4 Vocational Health College, Canakkale Onsekiz Mart University, Canakkale, Turkey; 5 Department of Developmental Neurobiology, National Institute of Perinatology, Lomas Virreyes, Mexico; 6 Veterans Affairs Normative Aging Study, Veterans Affairs Boston Healthcare System, Department of Medicine, Boston University School of Medicine, Boston, MA 02118, USA

**Keywords:** epigenetic, aging, mitochondria, telomere length, public health

## Abstract

Population aging is a looming global health challenge. New biological aging metrics based on DNA methylation levels have been developed in addition to traditional aging biomarkers. The prospective relationships of aging biomarkers with mitochondrial changes are still not well understood. Here, we examined the prospective associations of mitochondrial copy number (mtDNAcn) with several aging biomarkers – DNAm-Age, DNAm-PhenoAge, DNAm-GrimAge, and leukocyte telomere length. We analyzed 812 individuals from Veteran Affairs Normative Aging Study (NAS) with available blood samples from 1999-2013. Whole blood mtDNAcn and relative leukocyte telomere length were measured via qPCR. DNA methylation was assessed and used to calculate DNAm-Age, DNAm-GrimAge, and DNAm-PhenoAge. Linear mixed models were used to quantify the associations of mtDNAcn with DNAm-Age, DNAm-GrimAge, DNAm-PhenoAge, and leukocyte telomere length. In multivariable cross-sectional analyses, mtDNAcn is negatively associated with DNAm-Age PhenoAge and DNAm-PhenoAge. In contrast, mtDNAcn is associated with prospective measures of higher DNAm-PhenoAge and shorter leukocyte telomere length. Our study shows that higher mtDNAcn is associated with prospective measures of greater DNAm-PhenoAge and shorter leukocyte telomere length independent of chronological age. This indicates a role for mitochondrial in aging-related disease and mortality, but not the departure of biological age from chronological age.

## INTRODUCTION

In 2015, 8.3% of the global population was older than 65 years of age, but as the world population ages, this number is estimated to grow to 15.8% by 2050 [1]*.* Life expectancy is projected to continue to increase across the globe [2] and these trends have brought new public health challenges, such as the need to accurately measure the aging process and its associated health risks. Reliable biomarkers of aging may result in tools that can identify inter-individual differences in functional decline, disease onset, and mortality risk.

Telomere length is a well-known aging biomarker. Telomeres shorten with age in a broad range of organisms [3] and shorter telomere length has been associated higher rates of mortality from different age-related pathologies, including heart and vascular diseases, diabetes mellitus, Parkinson’s disease, and Alzheimer’s disease [4, 5]. More recently, the DNA methylation (DNAm) based estimator of biological age, DNAm-Age, has become another well-known molecular measure of human aging [6]. DNAm-Age has since been associated with cancers [6], cardiovascular diseases [7, 8], neurological diseases [9, 10], and chronic inflammation diseases [11]. A meta-analysis of 13 population-based cohorts including a total 13,089 individuals found that blood DNAm-Age was predictive of mortality, even when accounting for chronological age, concurrent diseases, and lifestyle risk factors [12], suggesting that DNAm-Age captures at least in part some additional aspect of biological aging. Subsequently, another DNAm based marker, DNAm-PhenoAge, was developed to be an improved predictor of mortality and health span [13] using phenotypic age estimated from a range of aging-related clinical measures. Most recently, another metric, DNAm-GrimAge [14], has been developed to predict all cause mortality and health span.

Unfortunately, the underlying biological and molecular processes that drive these epigenetic age biomarkers are still unknown. Indeed, there is still a lack of understanding about what they represent on a molecular level. A recent review [15] pointed out six major areas where relationships between DNAm-Age and molecular processes have been reported: cellular aging processes, nucleic acid processes, immune system processes, metabolic processes, cancer processes and animal models. Despite the observation that the DNAm-Age is associated with metabolic processes, the relationship between mitochondrial health and DNAm-Age remains understudied. Mitochondria are vital for metabolic processes as they are responsible for ATP production and are known to be involved in the aging process, become larger and less numerous with age, accumulating mutations, vacuoles, cristae abnormalities, and intramitochondrial paracrystalline inclusions [16, 17]. In addition, mitochondrial function may be related to DNAm aging. Activity of DNA methyltransferases (DNMT), as with any cellular enzyme, depend on ATP levels and impaired energy production as a result of mitochondrial dysfunction may influence normal function of DNMTs.

Mitochondrial DNA copy number (mtDNAcn), a measure of mitochondrial genome abundance, is commonly used as a reflection of the mitochondria’s response to oxidative stress as well as general dysfunction [18]. Mitochondria DNA (mtDNA) is sensitive to oxidative stress because it lacks a robust DNA repair system to restore oxidative stress induced damage and mtDNA damage persists longer compared to genomic DNA [19]. Typically, mtDNA will increase when the endogenous antioxidant response is no longer able to recover its redox balance [18], possibly as a compensatory response for insufficient ROS cleavage [20]. Previous studies have shown that mtDNAcn decreases with age [21–23] and is positively associated with telomere length [24–27]. Furthermore, mtDNAcn has been associated with several aging-related diseases such as various primary cancers [28], neurodegeneration [29], cardiovascular disease [30], and diabetes [18, 29].

Recently, our group has shown that cross-sectionally, mtDNAcn is negatively correlated with DNAm-Age and hypothesized mtDNAcn may be a proxy of mitochondrial buffer capacity [31]. Reduced mtDNAcn may be a consequence of exhausted mitochondrial buffering capacity, leading to adverse outcomes such as aging [31]. However, it is unknown whether mtDNAcn or this mitochondrial buffering capacity can predict accelerated biological aging. Thus, we extend upon our previous study and utilized data from the VA Normative Aging Study (NAS) cohort to examine the prospective associations of mtDNAcn with four aging biomarkers – DNAm-Age, DNAm-PhenoAge, DNAm-GrimAge and leukocyte telomere length.

## RESULTS

Table 1 describes the baseline characteristics of the participants in our study. For the 812 participants with available blood samples from 1999-2013, the mean chronological age at their first visit was 72.4 (±6.9) years. The majority of the subjects were former smokers (65.1%, n=529); consume fewer than two drinks/day of alcohol consumption (80.9%, n=657), were overweight (mean BMI 28.2, standard deviation (SD)=4.1), and have diagnosed hypertension (71.2%, n=578), but were free of coronary heart disease (70.7%, n=574) or diabetes (86.3%, N=701). Regarding mtDNAcn, expressed as a relatively ratio of total mtDNA copy numbers and nuclear DNA copy numbers, the mean was 1.0 (SD=0.3). For statistical modeling, we further divided mtDNAcn into quartiles. The mean baseline DNAm-Age, DNAm-PhenoAge, and DNAm-GrimAge were 73.0 (±7.9), 67.6 (±8.8), and 67.7 (±6.6) respectively**.** The mean leukocyte telomere length at baseline was 1.3 (±0.5). DNAm-Age was weakly correlated with DNAm-PhenoAge (r=0.33) and DNAm-GrimAge (r=0.29), but DNAm-PhenoAge and DNAm-GrimAge were strongly correlated (r=0.77) (Supplementary Figure 1). Telomere length was weakly correlated with DNAm biomarkers (r=-0.10 to -0.18).

**Table 1:** Baseline characteristics of normative aging study (NAS) participants in the current analysis (N=812).

	**N (%)**
Race	
White	791 (97.4%)
Non-White	20 (2.5%)
Smoking Status	
Current	36 (4.4%)
Former	529 (65.1%)
Never	247 (30.4%)
Alcohol Consumption	
<2 drinks/day	657 (80.9%)
≥ 2 drinks/day	155 (19.1%)
Hypertension	
Yes	578 (71.2%)
No	234 (28.8%)
Coronary Heart Disease	
Yes	238 (29.3%)
No	574 (70.7%)
Diabetes	
Yes	111 (13.7%)
No	701 (86.3%)
	Mean (SD)
Age (years)	72.4 (6.9)
BMI (kg/m^2^)	28.2 (4.1)
DNAm-Age (years)	73.0 (7.9)
DNAm-PhenoAge (years)	67.6 (8.8)
Relative Leukocyte Telomere Length	1.3 (0.5)
Relative Mitochondrial DNA Copy Number	1.0 (0.3)

Advancing chronological age was associated with lower mtDNAcn (β=-0.002; 95% confidence interval [CI]: -0.005, 0; p=0.05) and shorter leukocyte telomere length (β=-0.008;, 95% CI: -0.011, -0.004; p=0.05) and increased DNAm-Age (β=0.70; 95% CI: 0.64, 0.75; p <0.001), DNAm-PhenoAge (β=0.78; 95% CI: 0.72, 0.85; p <0.001), and DNAm-GrimAge (β=0.81; 95% CI: 0.77, 0.85; p <0.001) (Table 2).

**Table 2:** Cross-sectional associations of age with mitochondrial DNA copy number (mtDNAcn) and aging biomarkers.

	**mtDNAcn**		**DNAm-Age**		**DNAm-PhenoAge**		**DNAm-GrimAge**		**Telomere Length**	
	**β (95% CI)**	**p-value**	**β (95% CI)**	**p-value**	**β (95% CI)**	**p-value**	**β (95% CI)**	**p-value**	**β (95% CI)**	**p-value**
Age	-0.002	0.05	0.70	<0.001	0.78	<0.001	0.81	<0.001	-0.01	<0.001
	(-0.005, 0)		(0.64, 0.75)		(0.72, 0.85)		(0.77, 0.85)		(-0.01, 0)	

*Adjusted for smoking, alcohol use, BMI, cell composition, follow up time, hypertension status, CHD status, and diabetes status.

### Associations of mtDNAcn with cross-sectional measures of DNAm-Age, DNAm-PhenoAge, DNAm-GrimAge and leukocyte telomere length

**Figure 1:**
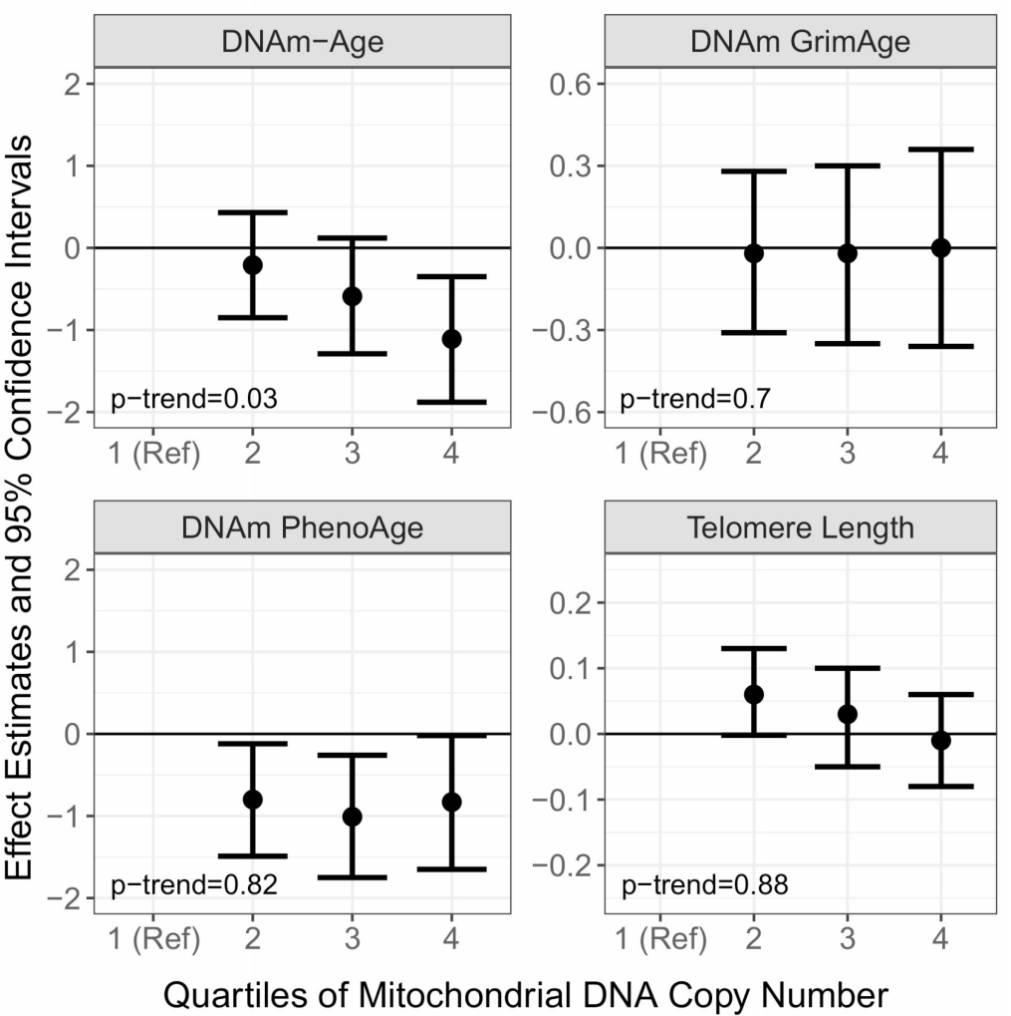
**Cross-sectional associations of Mitochondrial DNA Copy Number (mtDNAcn) with DNA Methylation Age (DNAm-Age), DNAm-PhenoAge, DNAm-GrimAge, and Telomere Length.** The effect estimates (β) and corresponding 95% confidence intervals were estimated with linear mixed models adjusted for chronological age, smoking, alcohol use, BMI, hypertension status, CHD status, diabetes status, blood cell type composition, and follow up time.

First, we sought to extend our previous analyses [31] using additional samples from 2011-2013 and to other aging biomarkers by examining the cross-sectional associations of mtDNAcn with DNAm-Age, DNAm-PhenoAge, DNAm-GrimAge, and leukocyte telomere length (Figure 1 and Supplementary Table 2). In multivariable models adjusting for chronological age, smoking, alcohol use, BMI, hypertension status, CHD status, diabetes status, blood cell type composition, and time since first visit, we observed a monotonic negative association between mtDNAcn and cross-sectional measures of DNAm-Age (p-trend=0.03). We also observed that compared to the lowest quartile of mtDNAcn, Q2 (β=-0.80; 95% CI=-1.49, -0.12; p=0.02), Q3 (β=-1.01; 95% CI=-1.75, -0.26; p=0.01), and Q4 (β=-0.83; 95% CI=-1.65, -0.02; p=0.04) of mtDNAcn were all negatively associated with DNAm-PhenoAge. However, there were no monotonic trends across these quartiles (p-trend=0.82). We did not observe associations of mtDNAcn with DNAm-GrimAge or leukocyte telomere length.

### Associations of mtDNAcn with prospective measures of DNAm-Age, DNAm-PhenoAge, DNAm-GrimAge and leukocyte telomere length

**Figure 2:**
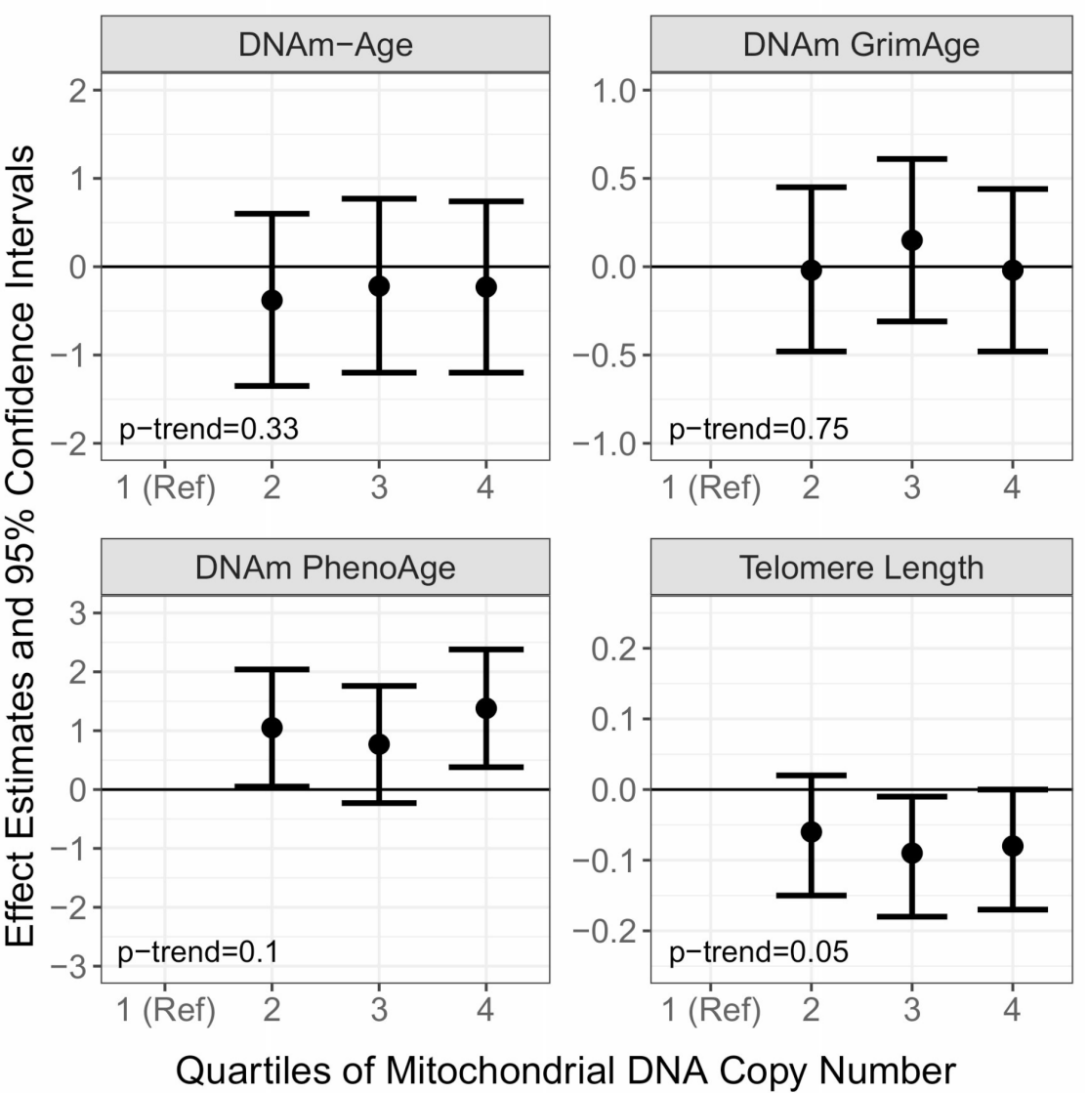
**Associations of baseline Mitochondrial DNA Copy Number (mtDNAcn) with prospective measures of DNA Methylation Age (DNAm-Age), DNAm-PhenoAge, DNAm-GrimAge, and Telomere Length.** The effect estimates (β) and corresponding 95% confidence intervals were estimated with linear mixed models adjusted for chronological age, measure at baseline, smoking, alcohol use, BMI, hypertension status, CHD status, diabetes status, blood cell type composition, and follow up time.

Next, we examined whether baseline mtDNAcn is associated with the aging biomarkers at all future visits by modeling the baseline measure of mtDNAcn with the aging biomarkers measured during subsequent follow-up visits (Figure 2 and Supplementary Table 3). In multivariable adjusted models, mtDNAcn was not associated with prospective measures of DNAm-Age (p-trend=0.33) or DNAm-GrimAge (p-trend=0.75). We did observe a positive association between mtDNAcn and prospective measures of DNAm-PhenoAge. Compared to the lowest quartile of mtDNAcn, Q2 (β=1.05; 95% CI=0.05, 2.04; p=0.04), Q3 (β=0.77; 95% CI=-0.23, 1.76; p=0.13), and Q4 (β=1.38; 95% CI=0.38, 2.38; p=0.01) were associated with higher DNAm-PhenoAge. However, there was no clear dose-response (p-trend=0.10). MtDNAcn was also associated with shorter leukocyte telomere length in subsequent visits (p-trend=0.05). Compared to Q1, Q3 (β=-0.09; 95% CI=-0.18, -0.01; p=0.03) and Q4 (β=-0.08; 95% CI=-0.17, 0.00; p=0.06) of mtDNAcn were both associated with shorter leukocyte telomere length.

### Sensitivity analyses

To investigate possible presence of reversal causality, we examined whether baseline DNAm-Age and baseline DNAm-PhenoAge were associated with prospective measures of mtDNAcn. There were no associations between baseline DNAm-Age and DNAm-PhenoAge with prospective measures of mtDNAcn (data not shown). Additionally, we tested the robustness of our models by excluding those who were diagnosed with CHD or diabetes, those who reported active smoking, or those who were not self-reported as white. In all cases, the resulting effect estimates were not meaningfully different compared to the results in Figures 1 and 2.

## DISCUSSION

In our analysis of 812 aging male veterans from the greater Boston area, we found contrasting results between cross-sectional and prospective analyses of mtDNAcn with aging biomarkers DNAm-Age, DNAm-PhenoAge, DNAm-GrimAge and leukocyte telomere length. Overall, we found suggestive evidence that mtDNAcn is positively associated with prospective measures of DNAm-PhenoAge and negatively associated with prospective measures of leukocyte telomere length. These observed associations were independent of chronological age, suggesting that higher mtDNAcn is associated to greater biological aging.

The results of our cross-sectional analyses presented here and in our previous report [31] are generally consistent with prior evidence. We observed that mtDNAcn is negatively associated with cross-sectional measures of DNAm-Age and DNAm-PhenoAge. This is consistent with expectations because mtDNAcn decreased with chronological age in both our study and in previous reports [21–23] while DNAm-Age and DNAm-PhenoAge expectedly increased with chronological age. Similarly, we observed that leukocyte telomere length decreased with chronological age in our study [32, 33], but we did not observe a positive association between mtDNAcn and leukocyte telomere length in our cross-sectional analyses as other cross-sectional studies have [24–27]. These differences in the telomere results may be a product of different study populations and different telomere length quantification methods.

Our prospective analyses stood in contrast to the cross-sectional results. Adjusting for measures of aging at baseline, mtDNAcn was positively associated with prospective measures of DNAm-PhenoAge. Similarly, although mtDNAcn was not associated with leukocyte telomere length cross-sectionally, there was an negative association between mtDNAcn and leukocyte telomere length at follow up visits, which is in accordance with studies that have previously reported that mtDNAcn is associated with shorter telomere length [34, 35]. These results suggest that while the negative cross-sectional associations reflect the opposing time-trends of mtDNAcn and aging biomarkers, it may be driven by unmeasured confounders such as underlying biological processes that drives both the decrease of mtDNAcn over time and the increase of DNAm-Age and DNAm-PhenoAge over time. By adjusting for baseline DNAm-PhenoAge and telomere length in our prospective analyses, we indirectly controlled for any underlying processes that would lead to changes in mtDNAcn and future DNAm-PhenoAge. There also the possibility that a cross-sectional analysis does not cover a sufficiently large time frame to detect modification of the aging biomarkers associated with mtDNAcn changes. It is notable that the relationship between mtDNAcn and DNAm-PhenoAge was not linear (p-trend=0.10) and not all quartiles reached statistical significance. Given that the effect estimates were all in the same direction, we speculate that this may reflect a non-linear relationship with a low threshold. Overall, our results suggest the mitochondria plays an active role in biological aging, with higher mtDNAcn leading to higher DNAm-PhenoAge and shorter telomere length independent of any underlying process that may cause both.

While we observed associations of mtDNAcn with DNAm-PhenoAge and leukocyte telomere length, no associations were observed with DNAm-Age and DNAm-GrimAge. This disparity may be due to the differences in the underlying biological drivers behind each of the aging metrics. DNAm-Age is based on chronological age and data obtained from multi-tissues samples and aims to reflect the general aging process. Specifically, it is thought that while DNAm-Age is associated with age-related diseases, it does not fully capture the risk differences for death and disease [13]. In contrast, DNAm-PhenoAge and DNAm-GrimAge were built on predictors of health and aim to better predict mortality from aging related diseases such as cardiovascular disease and cancer. These differences play an important role in terms of information that could be obtained from these metrics: DNAm-Age, being part of first-generation methylation based biomarkers, showed a strong correlation with chronological age but, with many age-related diseases and conditions, effects sizes of this correlation were small or moderate. As highlighted by Levine et al. a possible reason of this may reside in the fact that DNAm-Age does not include CpG sites that are important in terms of changes from physiological to pathological status [13]. This is why DNAm-PhenoAge was built using clinical biomarkers like albumin, creatinine, glucose serum, C-reactive protein, lymphocyte percent, white blood cell count, mean cell volume and others that have been shown to be better indicators of remaining life expectancy than chronological age [36]. DNAm-GrimAge was built from selected plasma protein biomarkers, smoking history, and time to death from all mortality causes and has been shown to be a better predictor of lifespan than previous DNAm-based predictors [14], but it is unclear whether this particular set of CpGs capture health and disease status in the same way as DNAm-PhenoAge. Shortened blood cell telomeres have been associated with higher rates of mortality from age-related pathologies [4, 5] and it is thought that shorter telomere length is not only due to progressing chronological time resulting in repeated cell replication, it also in part a result of the combined effects of oxidative stress and inflammation, two major components behind aging related diseases. Together, our data suggests that higher mtDNAcn may be associated with increased risk of aging-related disease and mortality, but not necessarily with departure of biological age from chronological age.

Currently, the underlying biological relationships between mitochondrial health, as reflected by mtDNAcn, and biological aging are unclear. It is possible that mitochondrial plays a role in aging-related biology and aging-related diseases either independently of mechanisms that affects both mitochondrial health and aging or as a mediator. Biologically, the mitochondrial genomic content (i.e. mtDNAcn) can increase in response to stress via mitochondrial biogenesis to enhance energy supply and repair damage to cellular components [37]. It is possible that increased energy due to increased mitochondrial genomic content may influence methylation status since more availability of energy means more ATP available for methylation/demethylation enzymes and their activity. This hypothesis is consistent with biological evidence that mitochondrial events can drive methylation profile in the nucleus [38]. Alternatively, because mtDNAcn has been associated with aging-related [18, 28–30], it is possible that the mtDNAcn impacts biological aging via roles in aging-related diseases. Lastly, mitochondria may drive DNA methylation changes in cellular entropy—characterized by less ATP production and higher heat dispersion [39]. The increased entropy of an aging cell may correlate to increased risk of aging related disease and mortality, reflected by an increase of an epigenetic metric like DNAm-PhenoAge

To our knowledge, our study is the first to report on the prospective associations of mtDNAcn with aging biomarkers DNAm-Age, DNAm-PhenoAge, DNAm-GrimAge and leukocyte telomere length. The current study possesses a number of strengths including the use of a large longitudinal cohort with repeated measures of aging and mitochondrial biomarkers. However, our study also has limitations. Our cohort is comprised primarily of white older men living in New England. There are other differences in the measures of biological aging by demographic factors (e.g. sex) that we were not able to examine. Thus, additional studies involving other demographic groups in different environments will be needed to confirm our findings more broadly. Finally, we used the existing literature and *a priori* knowledge of biological/clinical relevance to select and control for potential confounders, but we cannot rule out the possibility of unknown or residual confounding from underlying biological processes or external factors such as physical activity and psychosocial stress. In this scenario, mtDNAcn is a reflection or product of physical activity, psychosocial stress, or some underlying biological process that also causes biological aging or aging-related diseases and mtDNAcn may not be directly associated with higher DNAm-PhenoAge or lower leukocyte telomere length.

## CONCLUSIONS

Our study of 812 men from an aging cohort indicate the involvement of mitochondria in modulating biological aging, as reflected by DNAm-PhenoAge and leukocyte telomere length. Overall, we found evidence that higher mtDNAcn may be associated with higher DNAm-PhenoAge, indicating increased risk for aging-related disease and mortality, but not necessarily with departure of biological age from chronological age. Currently, the biological relationships between mitochondrial health and biological aging are not fully understood and future studies are necessary to further clarify the breadth of interactions between the mitochondria and aging biomarkers in human aging and to confirm our findings in other populations.

## MATERIALS AND METHODS

### Study population

The Veteran Affairs Normative Aging Study (NAS), a closed longitudinal cohort study of men from the Greater Boston area, was established in 1963 with 2280 individuals and followed up every 3-5 years. At enrollment, all participants were veterans 21-80 years of age, lived in the greater Boston area, and were free of chronic diseases. During follow-up study visits they undergo comprehensive outpatient medical evaluations and provide detailed data regarding diet and other lifestyle factors. Whole blood was collected after overnight fasting from each participant during NAS follow-up visits. The present study comprises 2186 visits from 812 subjects with available blood samples from 1999 to 2013 where 596 subjects had at least 1 follow-up visit and 438 had >2 follow-up visits.

### mtDNAcn measurement

Quantitative real-time polymerase chain reaction (qRT-PCR) was performed for mtDNAcn using total whole blood DNA as previously described [40, 41]. DNA samples were normalized and qRT-PCR was performed using primers listed in Supplementary Table 1. Nuclear DNA was quantified via TaqMan® RNase P Control Reagents Kit (Applied Biosystems). A laboratory reference DNA sample, which was a pool of 300 test samples (20 μL taken from each sample, final concentration: 40 ng/μL), was used to construct standard curves (mtDNA and nDNA R2 ≥0.99). The standard curves were used to quantify mtDNA and nDNA copy numbers to standardize the mtDNA/nDNA obtained from all test samples in all reactions [40]. We used mtDNA/nDNA in the statistical analysis. A ratio value of 1 indicates that the mtDNA/ nDNA of the test sample is equal to the mtDNA/nDNA in the reference DNA pool used in the assay. Each reaction was performed in triplicate and the mean was used for analysis. The within-run and between-run coefficients of variation of this assay were 3.35% and 3.26%, respectively [41].

### DNA methylation analyses and methylation clocks

DNA methylation from whole blood DNA extracted from the buffy coat layer was interrogated using the Infinium HumanMethylation450 BeadChip (Illumina). To minimize batch effects and ensure a similar age distribution across chips and plates, we randomized sample across plates and used a two-stage age-stratified algorithm to randomize samples. We pre-processed the samples with Illumina-type background correction without normalization, corrected for probe types using the BMIQ method [42], and removed probes below background fluorescence level (cutoff: p=0.05). For quality control, we removed samples where >5% of probes had a bead count <3 or if >1% of probes had a failed probe. DNAm-Age and DNAm-GrimAge were calculated using Horvath’s publicly available online calculator (https://dnamage.genetics.ucla.edu/home). The DNAm-PhenoAge has been calculated based on methods described by Levine et al. [13]. Lastly, white blood cell composition was estimated using the established reference-based method [43]

### Leukocyte telomere length assay

Leukocyte telomere length assay was performed using qRT-PCR as described [44–47]. In brief, buffy coat was obtained from whole blood samples and leukocyte DNA was purified using QIAamp DNA blood kit (Qiagen). DNA samples were normalized and qRT-PCR was performed using primers listed in Supplementary Table 1. To calculate relative leukocyte TL, we calculated the ratio of telomere repeat copy number (T) to human beta-globin copy number. To control for plate effects, leukocyte TL was expressed as the ratio between the leukocyte TL in the study sample vs. leukocyte TL from a DNA pool. This DNA standard pool was included on all PCR plates and consisted of DNA from randomly selected NAS participants and was used to create an eight-point standard curve, ranging from 0.234 to 30 ng/uL. The coefficient of variation for 15 test samples analyzed over 3 consecutive days was 8.7%, similar to the reproducibility originally reported for this method [46].

### Statistical analysis

A total of 812 participants had available blood samples from visits between 1999 and 2013. There were no differences in baseline characteristics between those included in our analyses compared to those excluded due to lack of biological samples. Outliers for mtDNA, DNAm-Age, DNAm-PhenoAge, and leukocyte telomere length were removed if the values were >3 SD from the mean.

We used random intercepts linear mixed models (LMMs) to study relationships mtDNAcn and the aging biomarkers. We first conducted cross-sectional analyses of mtDNAcn with aging biomarker outcomes where mtDNAcn from all visits were modeled with aging biomarkers from the same visits. Then, as prospective analyses, mtDNAcn from the first visit (i.e. earliest visit since 1999) was modeled as the exposure while the prospective measures of aging biomarkers from following visits were modeled as outcomes. For all models, mtDNAcn was modeled as both quartile and continuous variables. In the multivariable models, we controlled for the following covariates based on biological plausibility and previous works [31]: chronological age (continuous), follow up time (years, continuous), smoking status (never, current, former), cell type distribution (white blood cells, neutrophils, lymphocytes, monocytes, eosinophils, basophils, and platelets), alcohol consumption (<2drinks/day, ≥drinks/day), BMI (continuous), hypertension status (yes/no), diabetes status (yes/no), and coronary heart disease status (yes/no). For prospective analyses, baseline DNAm-Age, DNAm-PhenoAge, and leukocyte telomere length values were included in their respective models as covariates.

To examine the potential for reverse causation whereby greater biological aging may be driving mitochondrial changes, we examined the associations of baseline DNAm-Age, DNAm-PhenoAge, and leukocyte telomere length with prospective measures of mtDNAcn. For sensitivity analyses, we re-ran all analyses restricting to those without diabetes or CHD, non-active smokers, and only those who self-reported as white.

Analysis was performed with R (v3.5.2) (R Core Team (2013). R: A language and environment for statistical computing. R Foundation for Statistical Computing, Vienna, Austria.) using the ‘lme4’ package. Statistical significance was defined as p-values < 0.05.

## Supplementary Material

Supplementary Figure 1

Supplementary Tables
