# Correction for: Mitochondria and aging in older individuals: an analysis of DNA methylation age metrics, leukocyte telomere length, and mitochondrial DNA copy number in the VA normative aging study

**DOI:** 10.18632/aging.102962

**Published:** 2020-03-15

**Authors:** Jacopo Dolcini, Haotian Wu, Jamaji C. Nwanaji-Enwerem, Marianthi-Anna Kioumourtozlogu, Akin Cayir, Marco Sanchez-Guerra, Pantel Vokonas, Joel Schwarz, Andrea A. Baccarelli

**Affiliations:** 1 Department of Environmental Health Sciences, Mailman School of Public Health, Columbia University, New York, NY 10032, USA; 2 Department of Biomedical Sciences and Public Health, Section of Hygiene and Preventive Medicine, Medical School, Polytechnic University of Marche, Ancona, Italy; 3 Department of Environmental Health, Harvard T.H. Chan School of Public Health, Boston, MA 02115, USA; 4 Vocational Health College, Canakkale Onsekiz Mart University, Canakkale, Turkey; 5 Department of Developmental Neurobiology, National Institute of Perinatology, Lomas Virreyes, Mexico; 6 Veterans Affairs Normative Aging Study, Veterans Affairs Boston Healthcare System, Department of Medicine, Boston University School of Medicine, Boston, MA 02118, USA

**Keywords:** correction

Original article: Aging. 2020; 12:2070–2083.  https://doi.org/10.18632/aging.102722

**This article has been corrected:** The authors requested to correct the last name of Marianthi-Anna Kioumourtzoglou which was misspelled. This mistake does not change the content of publication.

The correct author’s name is provided below:

Jacopo Dolcini^1,2^, Haotian Wu^1^, Jamaji C. Nwanaji-Enwerem^3^, Marianthi-Anna **Kioumourtzoglou** ^1^, Akin Cayir^3,4^, Marco Sanchez-Guerra^5^, Pantel Vokonas^6^, Joel Schwarz^3^, Andrea A. Baccarelli^1^

