# Supplementary material for: Mitochondria and aging in older individuals: an analysis of DNA methylation age metrics, leukocyte telomere length, and mitochondrial DNA copy number in the VA normative aging study
Source: Aging (Albany NY). 2020 Feb 2;12(3):2070–83. doi: 10.18632/aging.102722 (PMC7041780; doi:10.18632/aging.102722)
Supplement: Supplementary Figure 1 [file aging-12-102722-s001..pdf]

SUPPLEMENTARY FIGURE

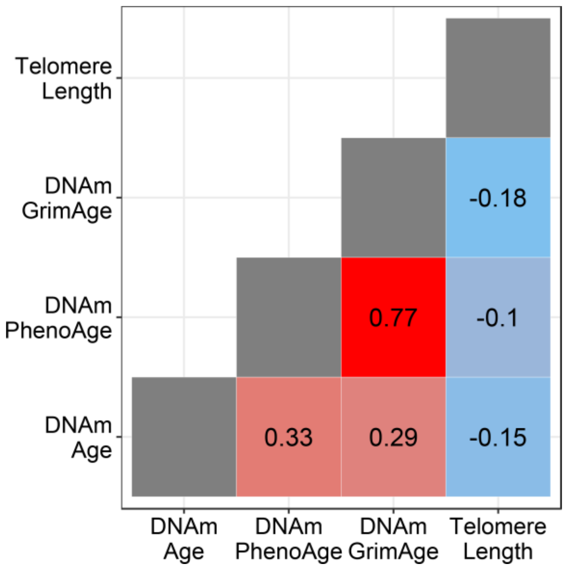

Supplementary Figure 1. Correlation matrix between the four aging biomarkers. The values in the figure show the coefficient estimates (rho) from Spearman correlation analyses for each estimate, also indicated by color. The p-values for all pairwise comparisons were <0.02.
