# Supplementary material for: Mitochondria and aging in older individuals: an analysis of DNA methylation age metrics, leukocyte telomere length, and mitochondrial DNA copy number in the VA normative aging study
Source: Aging (Albany NY). 2020 Feb 2;12(3):2070–83. doi: 10.18632/aging.102722 (PMC7041780; doi:10.18632/aging.102722)
Supplement: Supplementary Tables [file aging-12-102722-s002..pdf]

SUPPLEMENTARY TABLES

Supplementary Table 1. PCR primers.

Assay	Component	Sequence
mtDNA	mtDNA Forward Primer (mtF805)	5′ CCACGGGAAACAGCAGTGATT 3′
mtDNA	mtDNA Reverse Primer (mtR927)	5′ CTATTGACTTGGGTTAATCGTGTGA 3′
mtDNA	mtDNA TaqMan Probe (LifeTechnologies)	6FAM- 5′ TGCCAGCCACCGCG 3′-MGB
Telomere Length	Telc	5′-TGT TAG GTA TCC CTA TCC CTA TCC CTA TCC CTA TCC CTA ACA-3′
Telomere Length	Telg	5′-ACA CTA AGG TTT GGG TTT GGG TTT GGG TTT GGG TTA GTG T-3′
Telomere Length	Albd	5′-GCC CGG CCC GCC GCG CCC GTC CCG CCG GAA AAG CAT GGT CGC CTG TT-3′
Telomere Length	Albu	5′-CGG CGG CGG GCG GCG CGG GCT GGG CGG AAA TGC TGC ACA GAA TCC TTG-3′

Supplementary Table 2. Cross-sectional analysis of mitochondrial DNA copy number (mtDNAcn) and aging biomarkers.

mtDNAcn *	DNAm-Age		DNAm-PhenoAge		DNAm-GrimAge		Telomere Length	
	β (95% CI)	p-value	β (95% CI)	p-value	β (95% CI)	p-value	β (95% CI)	p-value
Q1	Reference		Reference		Reference		Reference	
Q2	-0.21 (-0.85, 0.43)	0.52	-0.80 (-1.49, -0.12)	0.02	-0.02 (-0.31, 0.28)	0.91	0.06 (-0.02, 0.13)	0.13
Q3	-0.59 (-1.29, 0.12)	0.10	-1.01 (-1.75, -0.26)	0.01	-0.02 (-0.35, 0.3)	0.90	0.03 (-0.05, 0.1)	0.49
Q4	-1.11 (-1.88, -0.35)	0.00	-0.83 (-1.65, -0.02)	0.04	0 (-0.36, 0.36)	1.00	-0.01 (-0.08, 0.06)	0.80
p-trend**	-1.47 (-2.79, -0.16)	0.03	-0.16 (-1.48, 1.16)	0.82	0.12 (-0.47, 0.7)	0.70	-0.01 (-0.11, 0.1)	0.88

*Models adjusted for chronological age, smoking, alcohol use, BMI, cell composition, follow up time, hypertension status, CHD status, and diabetes status.

Supplementary Table 3. Propsective analyses of baseline mitochondrial copy number (mtDNAcn) with aging biomarkers during follow up.

mtDNAcn	DNAm-Age		DNAm-PhenoAge		DNAm-GrimAge		Telomere Length	
	β (95% CI)	p-value	β (95% CI)	p-value	β (95% CI)	p-value	β (95% CI)	p-value
Q1	Reference		Reference		Reference		Reference	
Q2	-0.38 (-1.35, 0.6)	0.45	1.05 (0.05, 2.04)	0.04	-0.02 (-0.48, 0.45)	0.94	-0.06 (-0.15, 0.02)	0.16
Q3	-0.22 (-1.2, 0.77)	0.67	0.77 (-0.23, 1.76)	0.13	0.15 (-0.31, 0.61)	0.52	-0.09 (-0.18, -0.01)	0.03
Q4	-0.23 (-1.2, 0.74)	0.64	1.38 (0.38, 2.38)	0.01	-0.02 (-0.48, 0.44)	0.93	-0.08 (-0.17, 0)	0.06
p-trend**	-0.86 (-2.6, 0.87)	0.33	1.51 (-0.29, 3.31)	0.10	-0.22 (-1.64, 1.18)	0.75	-0.16 (-0.31, 0)	0.05

*All models adjusted for chronological age and outcome measure at baseline, follow up time, smoking, alcohol use, BMI, cell composition, hypertension status, CHD status, and diabetes status. **Using mtDNAcn as continuous models.
